# Display of individuality in avoidance behavior and risk assessment of inbred mice

**DOI:** 10.3389/fnbeh.2014.00314

**Published:** 2014-09-16

**Authors:** Torben Hager, René F. Jansen, Anton W. Pieneman, Suriya N. Manivannan, Ilan Golani, Sophie van der Sluis, August B. Smit, Matthijs Verhage, Oliver Stiedl

**Affiliations:** ^1^Sylics BVAmsterdam, Netherlands; ^2^Department of Functional Genomics, Center for Neurogenomics and Cognitive Research, VU University AmsterdamAmsterdam, Netherlands; ^3^Department of Clinical Genetics, VU University Medical CenterAmsterdam, Netherlands; ^4^Department of Molecular and Cellular Neurobiology, Center for Neurogenomics and Cognitive Research, VU University AmsterdamAmsterdam, Netherlands; ^5^Biobserve GmbHSt. Augustin, Germany; ^6^Department of Zoology, Faculty of Life Sciences and Sagol School for Neuroscience, Tel Aviv UniversityTel Aviv, Israel

**Keywords:** animal model, behavioral phenotyping, fear conditioning, individuality, learning, memory, passive avoidance, post-traumatic stress disorder

## Abstract

Factors determining individuality are still poorly understood. Rodents are excellent model organisms to study individuality, due to a rich behavioral repertoire and the availability of well-characterized isogenic populations. However, most current behavioral assays for rodents have short test duration in novel test environments and require human interference, which introduce coercion, thereby limiting the assessment of naturally occurring individuality. Thus, we developed an automated behavior system to longitudinally monitor conditioned fear for assessing PTSD-like behavior in individual mice. The system consists of a safe home compartment connected to a risk-prone test compartment (TC). Entry and exploration of the TC is solely based on deliberate choice determined by individual fear responsiveness and fear extinction. In this novel ethological assay, C57BL/6J mice show homogeneous responses after shock exposure (innate fear), but striking variation in long-lasting fear responses based on avoidance and risk assessment (learned fear), including automated stretch-attend posture quantification. TC entry (retention) latencies after foot shock differed >24 h and the re-explored TC area differed >50% among inbred mice. Next, we compared two closely related C57BL/6 substrains. Despite substantial individual differences, previously observed higher fear of C57BL/6N vs. C57BL/6J mice was reconfirmed, whereas fear extinction was fast and did not differ. The observed variation in fear expression in isogenic mice suggests individual differences in coping style with PTSD-like avoidance. Investigating the assumed epigenetic mechanisms, with reduced interpretational ambiguity and enhanced translational value in this assay, may help improve understanding of personality type-dependent susceptibility and resilience to neuropsychiatric disorders such as PTSD.

## Introduction

Individuality is commonly defined as the collection of divergent behavioral and physiological traits among individuals and develops when unique environmental influences act on the genome, following complex routes, to produce phenotypic diversity (Champagne, 2013). Consequently, enrichment experiments demonstrate that experience contributes to the development of individuality and affects behavioral performance (Rosenzweig and Bennett, 1996). Emergence of individuality is under intense investigation, as it is considered central in the development of several neuropsychiatric disorders. Personality-type differences are suspected to be predictive of disease incidence, progression and recovery, for instance in major depression and posttraumatic stress disorder (Zovkic and Sweatt, 2013). With some exceptions (Freund et al., 2013), display of individuality and personality type dichotomies have been hard to model using animal models. In classical behavior tests individual variation, the quantitative measure for observed differences of a particular phenotype, is considered a disadvantage since it negatively affects statistical power and replicability (Button et al., 2013). However, animal models offer unique advantages to study individuality due to the availability of well-characterized inbred populations. Furthermore, systematic analysis of individuality offers the potential to exploit behavioral extremes that could serve as improved disease/disorder models (Lathe, 2004; Borsini, 2012).

Commonly used test systems are not tailored to measure inter-individual differences and are in fact often unsuited to assess individual variation. First, behavioral phenotypes of rodent models are typically acquired in a novel test environment. This introduces coercion (stressor), which is a confounding factor, particularly when the emotional state of animals is investigated (Hurst and West, 2010). Hence, most experiments are confounded by the susceptibility of animals to experimenter-based perturbation of cognitive function (Diamond et al., 2007), instead of investigating intrinsically motivated performance to challenging conditions. Second, these tests are typically of short duration. The assessment of individuality clearly benefits from measuring behavior on appropriate time-scales (Fonio et al., 2012a,b). Third, classical behavior tests mainly quantify a single measure, which limits the analysis of complex behaviors and individuality. Integration of multiple measures may be particularly advantageous when it concerns learning, memory, and emotional states (Koolhaas et al., 2006). Additionally, freezing—the classical behavioral fear measure in mice—is of limited symptomatic relevance in humans (Azevedo et al., 2005; American Psychiatric Association, 2013). Although freezing is a sensitive measure of fear, it was claimed that it may be only measurable during the state of fear (Lang et al., 2000) and it is unsuited under conditions of active coping (flight or escape). The progression of other unambiguous fear-related measures that can only be acquired when the mouse is observed over longer time intervals might yield information that is more relevant to the human phenotype.

Automated analysis of behavior in a home cage design solves many of the limitations outlined above. Various solutions exist for automated tracking and analysis (de Visser et al., 2006; Kas et al., 2008, 2011; Fonio et al., 2009; Urbach et al., 2014). We developed a flexible modular system (“DualCage”) that consists of a home compartment (HC) and attached test compartment (TC). This system combines the advantage of uninterrupted long-term monitoring without human intervention in the home cage setup (de Visser et al., 2006; Steele et al., 2007; Jhuang et al., 2010; Voikar et al., 2010; Viviani et al., 2011; Maroteaux et al., 2012) with the assets of deliberate exploration of an attached novel environment (Fonio et al., 2009). In the DualCage system the animal has, to a certain extent, the choice to deliberately participate in an experiment, and the progression of the experiment is determined by the instrumental responses of the animal. This offers improved ethological relevance, i.e., the responses are biologically meaningful, by taking species- and strain-specific characteristics into account (Belzung and Griebel, 2001; Olsson et al., 2003). The assessment of multiple behavioral measures based on the 3-point tracking in the DualCage enabled us to delineate risk assessment and avoidance (Augustsson and Meyerson, 2004; Blanchard et al., 2011). The stretch-attend posture (Grant and Mackintosh, 1963) is an important activity-independent behavioral expression in the context of risk assessment for detection and analysis of threat stimuli (Stankowich and Blumstein, 2005; Blanchard et al., 2011). An extended memory test with a duration of 2 days allowed revealing inter-individual differences in fear responsiveness through different coping styles (Koolhaas et al., 2007) in isogenic mouse lines (Freund et al., 2013) with enduring conditioned fear responses. The discriminative power of the DualCage system became apparent by comparing the genetically closely related substrains C57BL/6J and C57BL/6N, which, despite genetically differing only by a relatively low number (~20) of single nucleotide polymorphisms (Petkov et al., 2004; Mekada et al., 2009; Zurita et al., 2011), differ in their fear responses (Radulovic et al., 1998; Stiedl et al., 1999; Siegmund et al., 2005; Bryant et al., 2008).

## Materials and methods

### Animals

Mice were obtained at an age of 8 weeks and individually housed upon arrival at constant 12-h dark-light cycle under controlled temperature (21 ± 1°C) and humidity (55 ± 10%) conditions with *ad libitum* access to food and water. Experiments started after an acclimation period of 1–2 weeks to the housing facility with shifted 12-h dark-light cycle (lights off at 2 p.m.). The illuminance during the light phase was approximately 40 lx, whereas red light was provided during the dark phase. Mice were 9–11-weeks-old during the experiment. Data from 24 male C57BL/6JIco and 27 male C57BL/6NCrl mice (Charles River, Netherlands) were analyzed in these experiments. All studies involving animals were approved by the animal research committee of the VU University Amsterdam according to Dutch regulations and comply with the European Council Directive (86/609/EEC).

### DualCage

**Figure 1:**
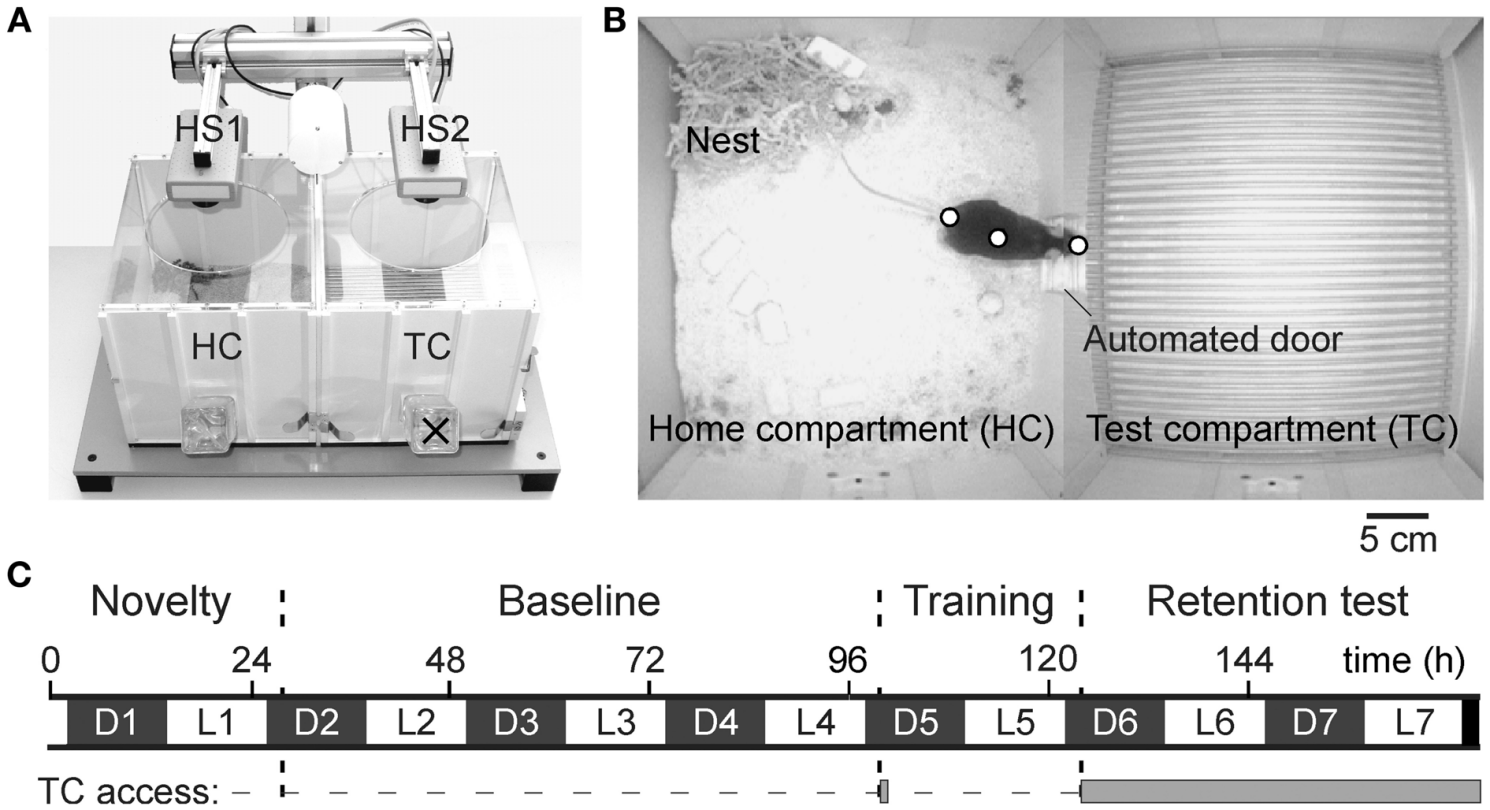
**DualCage design and experimental procedure. (A)** Picture of the DualCage (frontal top view) with the two head stages (HS1 and HS2) video-tracking the mouse in both home (HC) and test compartment (TC). Please note that no bottle was provided in the TC (×) in the present experiments. **(B)** Merged video frames of both cameras monitoring HC and TC with the software-controlled door being open. The three body points, tail base, center of gravity and nose tip that are depicted on the mouse, were used for tracking. **(C)** Experimental sequence along the 7 days of an experiment with the different tests and the dark (D1–D7) and light phases (L1–L7) indicated. The solid gray bar indicates the access (open door) to the TC. Fear-conditioned but not control mice were subjected to a single foot shock 30 s after the TC entry during training (D5).

The automated home cage environment (DualCage; commercially available as *HomeCage*^Plus^, Biobserve, St. Augustin, Germany; Figures 1A,B) consists of a home compartment (HC; 30 × 25 × 23 cm, width × depth × height) attached to the TC separated by a sliding door (5 × 6 cm, width × height). A control unit with USB-TTL I/O connection provided for hardware control.

A separation of home and test compartment is important to exploit a conflict situation between novelty-seeking and avoidance behavior of mice at distinct times after training to investigate short- and long-term fear memory similar to the passive avoidance test (Ögren and Stiedl, 2010). Two cameras tracked the behavior in a region of interest of 320 × 264 pixels for each compartment. The rear zone (10 cm) of the HC was excluded from the body length analysis, because here the nest site of mice was located in all cases which, based on the curled body posture and cover by nesting material, confounded the correct body length measurements. Few shocked mice that initially built their nest close to the door “moved” it to the rear wall predominantly upon reopening of the door in the retention test indicating an active coping strategy to increase the distance to the TC. By using a 2-channel frame grabber both camera images were merged resulting in one video image for data analysis. Data were assigned to different groups after the export of batch-processed raw data based on customized software (Viewer©, Biobserve GmbH, St. Augustin, Germany). Short-term artifacts in the 3-point-tracking, caused by a frame-to-frame inversion of nose and tail tracking position, were automatically detected and corrected by a low-pass filter within a period of less than 400 ms (10 frames) in the offline analysis and replaced with neighboring values. Long-term artifacts caused by an inversion of nose and tail at low frequency could not be detected in this automated manner. These artifacts occurred during episodes of rearing and sleep and were irrelevant to the behavior responses analyzed here. Body length tracking was not confounded during crucial behaviors such as risk assessment based on stretch-attend postures in the door region. Cages for tests with or without shock exposure were randomly chosen among 10 available DualCages and alternated across test batches according to k-permutation.

The behavior was monitored by two cameras using specific tracking software (Viewer©, Biobserve). A Viewer© software script controlled all hardware actions in an operant fashion. The behavior was monitored for 7 days during light and dark phases (Figure 1C) and the video was stored digitally. Mice were not handled during the whole experimental time. Infrared diodes provided for a constant illumination undetected by mice. Specific camera filters allowed optimal tracking throughout the circadian cycle. The digitally recorded video material (recorded by the Viewer© tracking software at ~25 Hz) was crucial for quality control of automated tracking data and recheck of specific behavioral responses. Posture analysis was performed on the basis of a three-point detection algorithm (Viewer©, Biobserve) that allowed recognizing the nose tip, the center of gravity and the tail base of a mouse.

### Experimental training sequence and behavioral measures

The experimental sequence is depicted in Figure 1C. Initially, 1 day after placement in the HC, baseline HC behavior was monitored for 3 days. Contextual fear conditioning training was initiated after the onset of the dark phase of day 5 by automatically opening the door to the TC compartment. Upon full entry of the TC each mouse was confined for 30 s and then exposed to a single shock (US: unconditioned stimulus; 0.7 mA, 2 s, scrambled) whereas control mice did not receive a shock. After US offset, the door was opened and upon HC return, the door was closed again for ~24 h until it was reopened 1 h after the onset of the dark phase of day 6 and then remained open for 2 days. The time to open the door was chosen according to the highest level of mobility as indicated by circadian activity (de Visser et al., 2006) and running-wheel activity of B6J mice (e.g., de Visser et al., 2007; Rosenwasser and Fixaris, 2013).

All behavioral measures were based on 3-point tracking of mice. The circadian activity of mice was monitored based on the center of gravity. Operational definitions of all measures are provided in Table 1. The progression of HC and TC exploration was determined by the Boolean map of exploration (Figure S1). Both TC and HC area were segmented into 5 × 5 pixel zones so that the total number of zones (~3000) corresponds to 100% of accessible area of each compartment. The first visit of each zone determined by the nose tracking point of a mouse was cumulatively quantified in a binary manner (visited/not visited). Thereby, a mouse that fully explores the TC or HC will eventually reach the maximum value of 100% explored area. In addition, visits of the door area (Table 1) were determined for a half-circular zone with a maximum distance of 3 cm from the door on the basis of the nose tip of mice. The body length of mice was determined as a function of distance to the door. These values were plotted for the first 15 min of the retention test and a linear fit was used to determine the slope.

**Table 1:** **Operational definitions of behavioral expressions and measures**.

**Measure**	**Definition**
Door approach	Nose tip in a half-circular door area (distance to door max. 3 cm) in the HC
Peeking	Tail base and center of gravity in HC or TC, nose tip in the other compartment
Partial entry	Tail base in HC or TC, center of gravity and nose tip in the other compartment
Full entry	All three body points tracked in one compartment
Exploration	Percentage of Boolean map area^*^ explored by the nose tip within HC and TC

HC, home compartment; TC, test compartment;

* *see Figure S1*.

### Data analysis

Behavioral differences were examined on the basis of analysis of variance (ANOVA), repeated measures ANOVA or nonparametric comparison by Mann–Whitney U-test when appropriate. Transfer latencies were plotted as cumulative incidence of transfer and compared by Cox regression (Jahn-Eimermacher et al., 2011). For better comparability, we determined the *T*_50_-values, i.e., the values when 50% of the mice entered the TC. Pearson's *r* rank correlation coefficients (z-score transformation) were computed using algorithms as described by Press et al. (1988) in the correlation matrix. An error probability level of *P* ≤ 0.05 corrected by false discovery rate (FDR) analysis was accepted as statistically significant throughout the study. To correct for potentially inflated type I error due to multiple comparisons *P*-values were corrected by the minimum positive FDR. We followed a previously reported procedure (Verhoeven et al., 2005) with a threshold set at 5% detecting one potential type I error in the correlation matrix of extracted measures (**Figure 5**). Analyses were performed using StatView 5.0.1 and JMP 5.0.1a (SAS Institute, Cary, NC, USA). Non-parametric data are presented as box plots with the ends of the box denoting the 25 and 75% interquartile range and the whiskers providing the upper and lower quartile ± 1.5 times the interquartile range, respectively, while the line in the box denotes the median.

## Results

### Exploration and immediate stress responses are homogeneous among C57BL/6J mice

**Figure 2:**
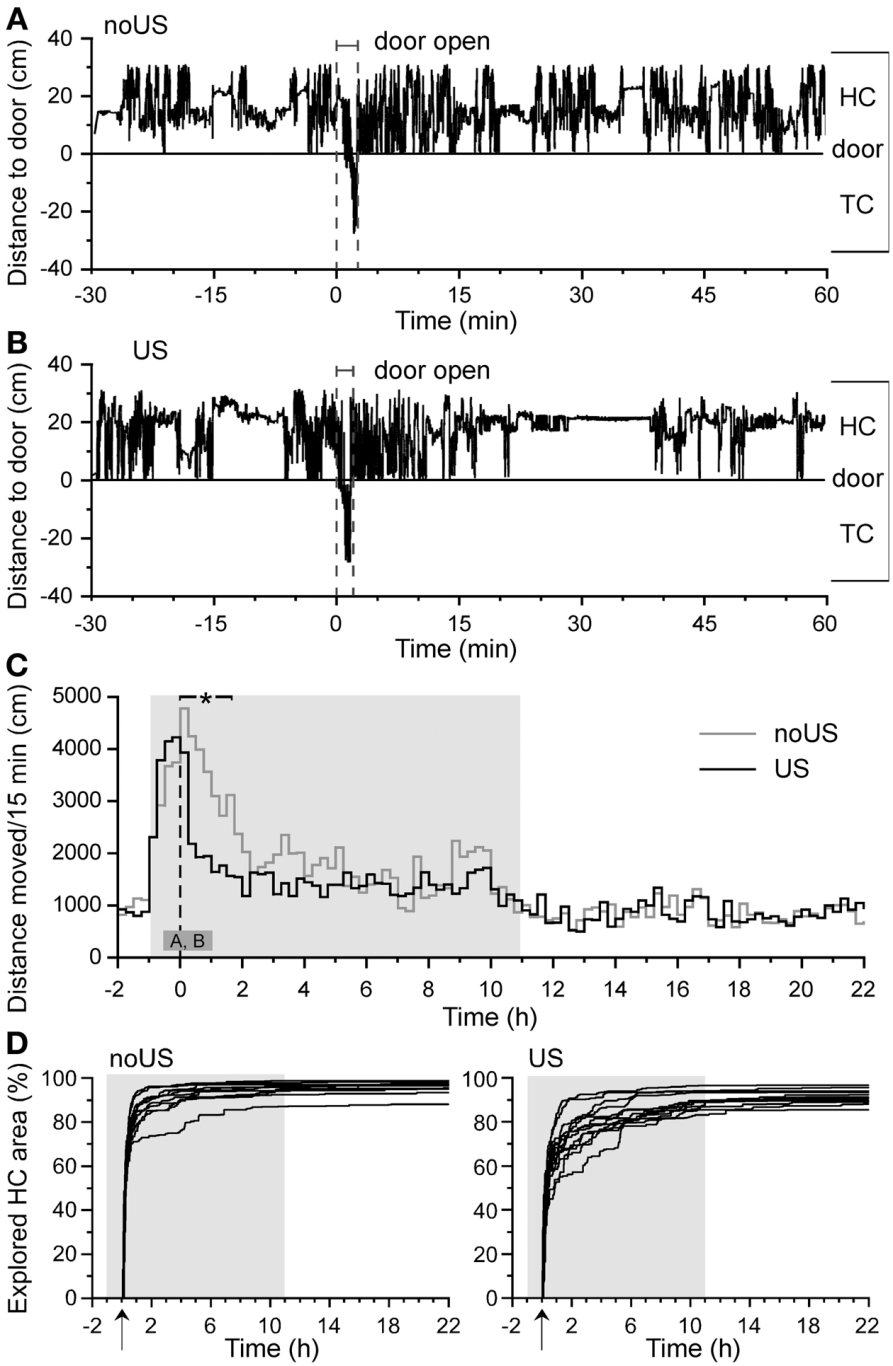
**Training-related behaviors of C57BL/6J mice indicate fast TC entry (low anxiety) and post-shock activity suppression (unconditioned stress)**. Typical activity patterns of **(A)** a non-shocked (noUS) and **(B)** a shocked mouse (US) with its distance to the door in HC and TC from 30 min before to 60 min after the TC entry. The negative deflection denotes the brief TC entry during training ± shock exposure. **(C)** Mean activity of non-shocked (noUS; *n* = 11) and shocked mice (US; *n* = 13) throughout the training day with the dashed vertical line indicating when the door to the shock compartment was opened. The small dark gray block indicates the time of the two panels shown above **(A,B)**. The bar on the top denotes the range of significant activity differences [*F*_(1,22)_ ≥ 5.29; ^*^P < 0.05]. The door was opened *X* = 0 min/h or as indicated by an arrow, gray background areas denote dark phases. After HC return from the TC after training, there was a slower home cage re-exploration in shocked than in non-shocked C57BL/6J **(D)** mice as determined by the Boolean map of progressive exploration.

To assess variation in long-term fear responses of individual mice, B6J mice were placed in the HC of the DualCage 1 h before onset of the dark phase. Thereafter, mice habituated to the HC for 3 days. The door to the TC was opened 1 h after the onset of the dark phase of day 5 to start fear conditioning training. All mice entered the TC spontaneously with a latency of less than 2 min (see **Figure 4A**). After the full TC entry the door closed to confine the mouse in the TC. Thirty seconds after closing, either a shock (unconditioned stimulus) or no shock was given (control). Thereafter the door was reopened. All mice (shocked and non-shocked) returned to the HC immediately (Figures 2A,B; HC return time 12.1 ± 2.0 s, mean ± SEM) without significant difference between groups and the door to the TC was closed.

After training, mice that had received a foot shock were significantly less active in the HC than non-shocked mice (Figure 2C). This difference emerged immediately in the HC [*F*_(1,22)_ = 22.03; *P* < 0.0001] and lasted up to 1.75 h after shock exposure [*F*_(1,22)_ = 8.12; *P* = 0.0093]. Activity measurements in 1-s bins from 10 s before to 10 s after the 2-s shock exposure in the TC during training were used to identify the significant shock-induced activity increase [~5-fold compared to basal activity; *F*_(1,22)_ = 233.50 and 61.11, respectively; *P* < 0.0001] compared with non-shocked control mice. There was no difference in the shock-induced activities of B6J and B6N mice suggesting similar pain perception and response (data not shown). Shocked mice re-explored their HC after return from the first TC visit with a slower progression and slightly lower total area than control mice (Figure 2D, Figure S2C). Especially shock-exposed mice spent significantly less time in the door area than non-shocked mice (Figure S2D). These observations indicate that naïve habituated mice show low variation in their latency to enter the novel environment (TC), in their latency to return to the HC, and in their typical fear-induced activity suppression after shock exposure, consistent with corresponding measures in classical fear assays (**Figure 6**).

### Posture differences are powerful indicators of avoidance behavior

**Figure 3:**
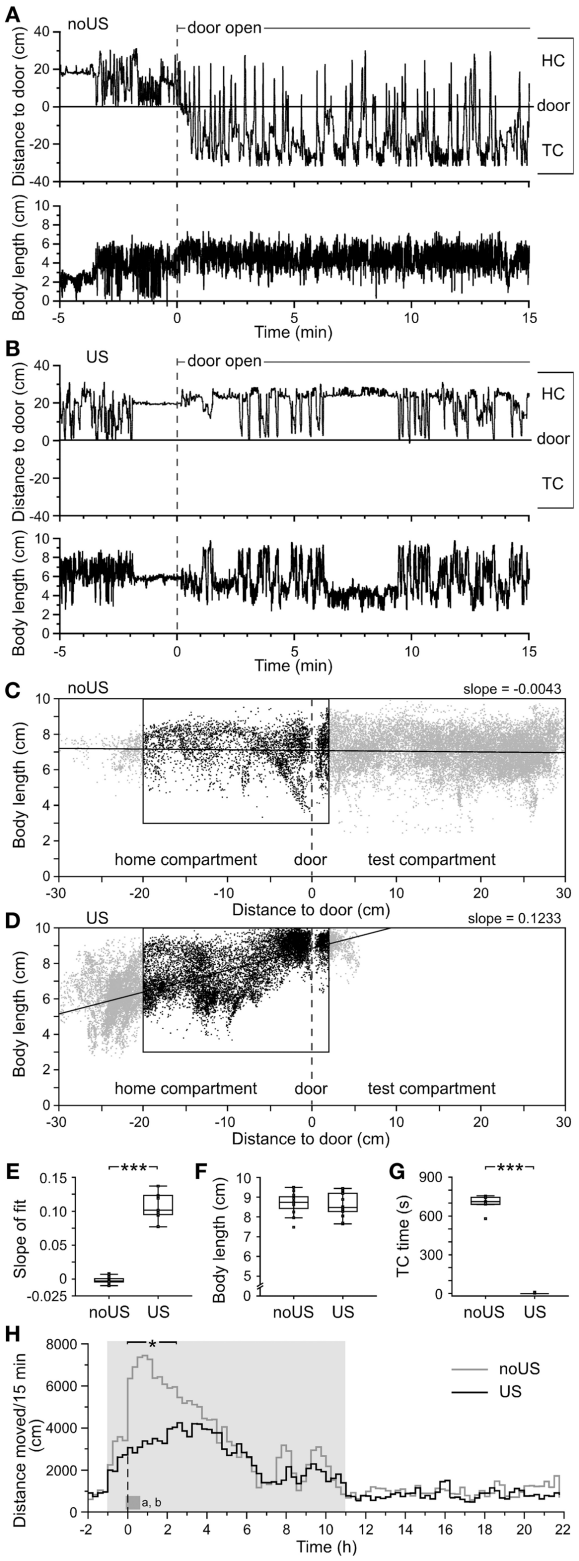
**Retention test behaviors indicate increased risk assessment and avoidance in fear-conditioned vs. control C57BL/6J mice**. The position of the nose tip is plotted together (top panel) with the body length (lower panel) in HC and TC for 5 min before and 15 min after opening the door to the TC for **(A)** a non-shocked (noUS) and **(B)** a shocked mouse (US). The non-shocked mouse maintained an intermediate body length irrespective of position in HC and TC. In contrast, the shocked mouse alternated between the rear end of the HC and door approaches (including one partial TC entry) with highly variable body length. Short body length values of mice, particularly before door opening, are related to rearing. The changes in distance to door correlated with locomotor activity (data not shown). **(C)** The plot of body length vs. distance to the door of the non-shocked mouse showed a relatively homogeneous intermediate body length irrespective of its position in both HC and TC, whereas the shocked mouse **(D)** showed a more irregular pattern with reduced body length at larger distance from the door and increased body length toward the door. **(E)** The slopes of the least square linear fits during the first 15 min with reopened door showed a significant difference between non-shocked and shocked mice. **(F)** In naïve mice, the body length did not differ during the TC entry in the training. **(G)** Significant difference in the time spent in the TC during the first 15 min after door opening. **(H)** Mean activity of noUS and US mice throughout the retention test day with the dashed vertical line indicating when the door to the shock compartment was opened. Time (*X* = 0 min) indicates opening of the door. The dark gray block indicates the time of the two panels shown above **(A,B)**. The bar on the top denotes the 2.5-h range of significant activity differences [*F*_(1,22)_ ≥ 5.35; ^*^P < 0.05]. noUS: *n* = 11; US: *n* = 13; ^***^P < 0.0001 (Mann–Whitney U-test).

Twenty-four hours later (day 6; Figure 1C), again 1 h after the onset of the dark phase, the door reopened and remained open for 48 h to determine fear memory (retention) and extinction performance. During the first hour of the dark phase of day 6, 23–24 h after the shock, shock-exposed B6J mice did not differ from non-shocked B6J mice in locomotor activity (Figures 3A,B,H) providing no indications of generalized fear when the door was still closed. However, when the door was reopened, non-shocked mice immediately re-entered the TC (Figure 3A), whereas shocked mice did not (Figure 3B). Opening of the door instantly triggered increased alertness in shocked mice indicated by changes of body posture, avoidance of the door area, and reduced exploratory behavior (Movie S1). Non-shocked mice had a relatively constant body length (mean ~7 cm) irrespective of their location in either HC or TC (Figure 3A). In contrast, shocked mice showed freezing in a crouched posture (reduced body length) in the rear part of the HC and infrequent approaches toward the door, increasing their body length due to the stretch-attend posture (maximum ~10 cm) (Figure 3B and Movie S1). The relation of body length vs. nose position in HC and TC (Figures 3C,D) indicated highly significant body posture differences (slope differences: *U* = 143; *P* < 0.0001) in shocked vs. non-shocked mice in the first 15 min after reopening the door (Figure 3E). Especially when approaching the door, shocked mice showed a significantly increased body length (Figure 3D). In contrast, no body length difference existed between the two groups during training (Figure 3F).

### Individuality among C57BL/6J mice emerges in long-term fear responses

**Figure 4:**
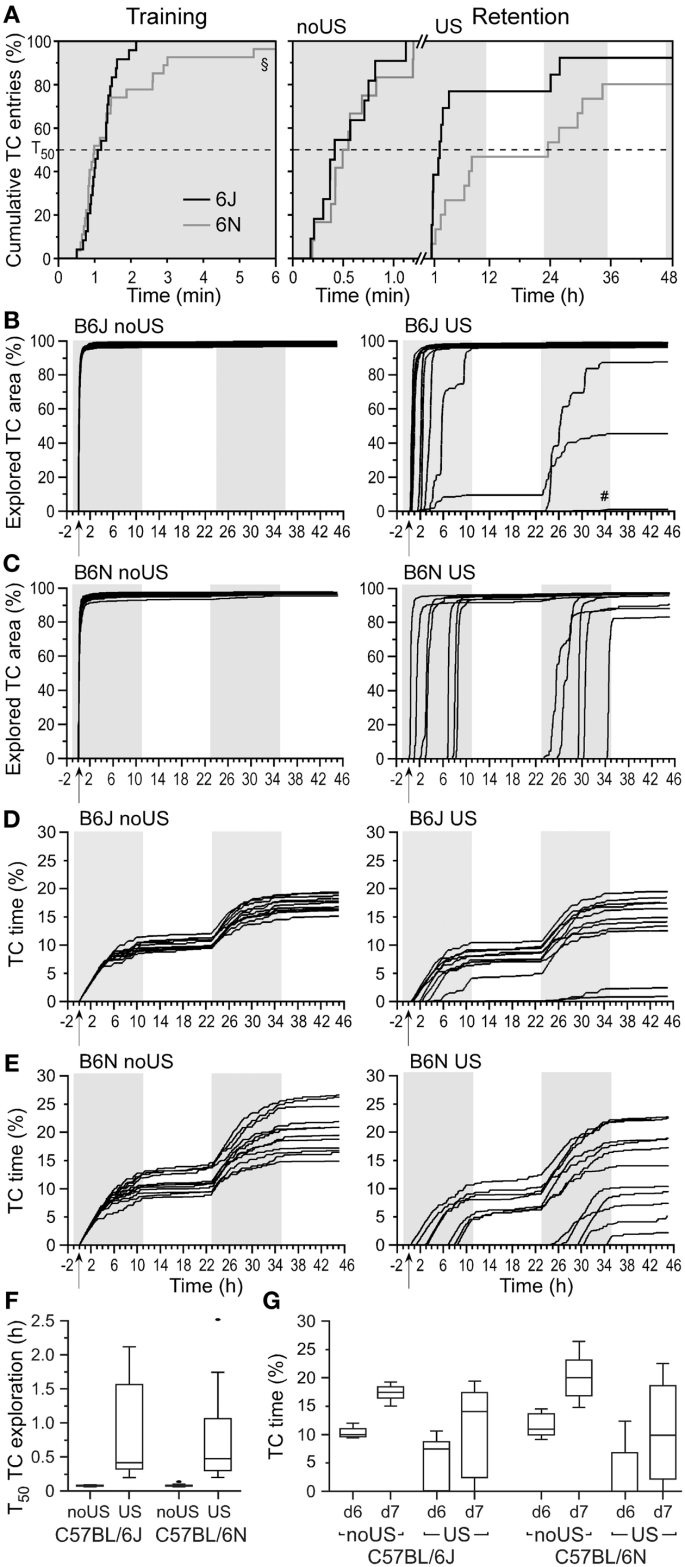
**Transfer latencies differ between fear-conditioned C57BL/6J (B6J) and C57BL/6N (B6N) mice with stronger avoidance (fear) in B6N than in B6J mice followed by similar TC exploration (fear extinction). (A)** Transfer latencies for the first full TC entry based on the cumulative incidence of transfer plot of pooled non-shocked (noUS) and shocked (US) mice during training, and retention test of noUS and US mice indicating a significant difference between shocked B6J and B6N mice in retention. Each vertical step with the amplitude of 100/*n* (%) denotes the response of one individual. *T*_50_ indicates when 50% of the mice of each group entered the TC. Note that 1 out of 13 B6J and 3 out of 15 B6N mice did not re-enter the TC within 48 h. Boolean map of progressive TC exploration of non-shocked (noUS) and shocked (US) B6J mice **(B)** indicated an instant onset and fast exhaustion of TC exploration in all mice after the first full entry and delayed onset of exploration with generally slightly lower slope except for four B6J mice with delayed progression (^#^denotes low values of one mouse). **(C)** The TC exploration in non-shocked B6N mice (no US) was similar to that of B6J mice. Shocked B6N mice showed a delayed TC entry but relatively fast TC exhaustion. The cumulative time spent in the TC in B6J **(D)** and B6N mice **(E)** shows similar progression after its start and corresponds to the explored TC area. Quantitative comparison of both substrain performances is shown for the halftime (*T*_50_) to explore 50% of the TC after its first full entry (from **B** and **C**) in the retention test **(F)** that depends on shock exposure but not substrain. The total time spent in the TC **(G)** during the first (d6) and the second day (d7) of the retention test (from **D** and **E**) are lower in shocked mice with higher variation. Arrows denote the opening of the door; gray background areas denote dark phases. B6J noUS: *n* = 11; B6J US: *n* = 13; B6N noUS: *n* = 12; B6N US: *n* = 15.

**Figure 5:**
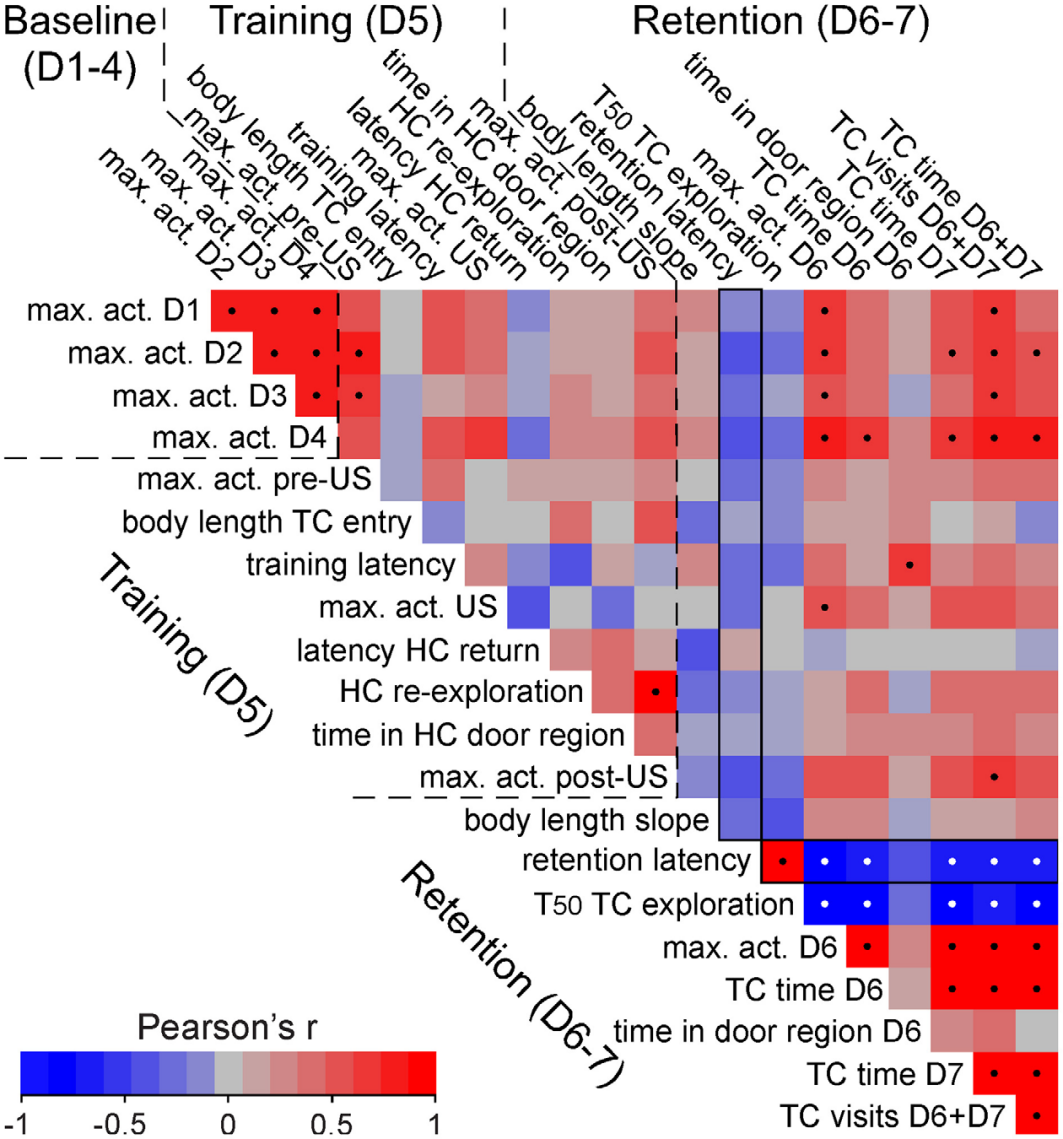
**Correlation matrix of behavioral performances of shocked C57BL/6J mice**. High correlation of activity data across days but lack of correlation with increased retention test transfer latencies in shocked C57BL/6J mice. Correlation matrix of different behavioral measures based on Pearson's linear correlation. The order of measures follows the time line of the DualCage measures from baseline via training to retention test. Black and white dots indicate significant correlations after FDR correction. The exemplified correlation plot shows that the retention latency is positively correlated with the halftime (*T*_50_) of TC exploration. In contrast, no significant correlation was found in baseline and training measures with the retention latency (framed vertical column). act., activity; D, dark phase; HC, home compartment; max., maximum; TC, test compartment; train., training; US: shock.

**Figure 6:**
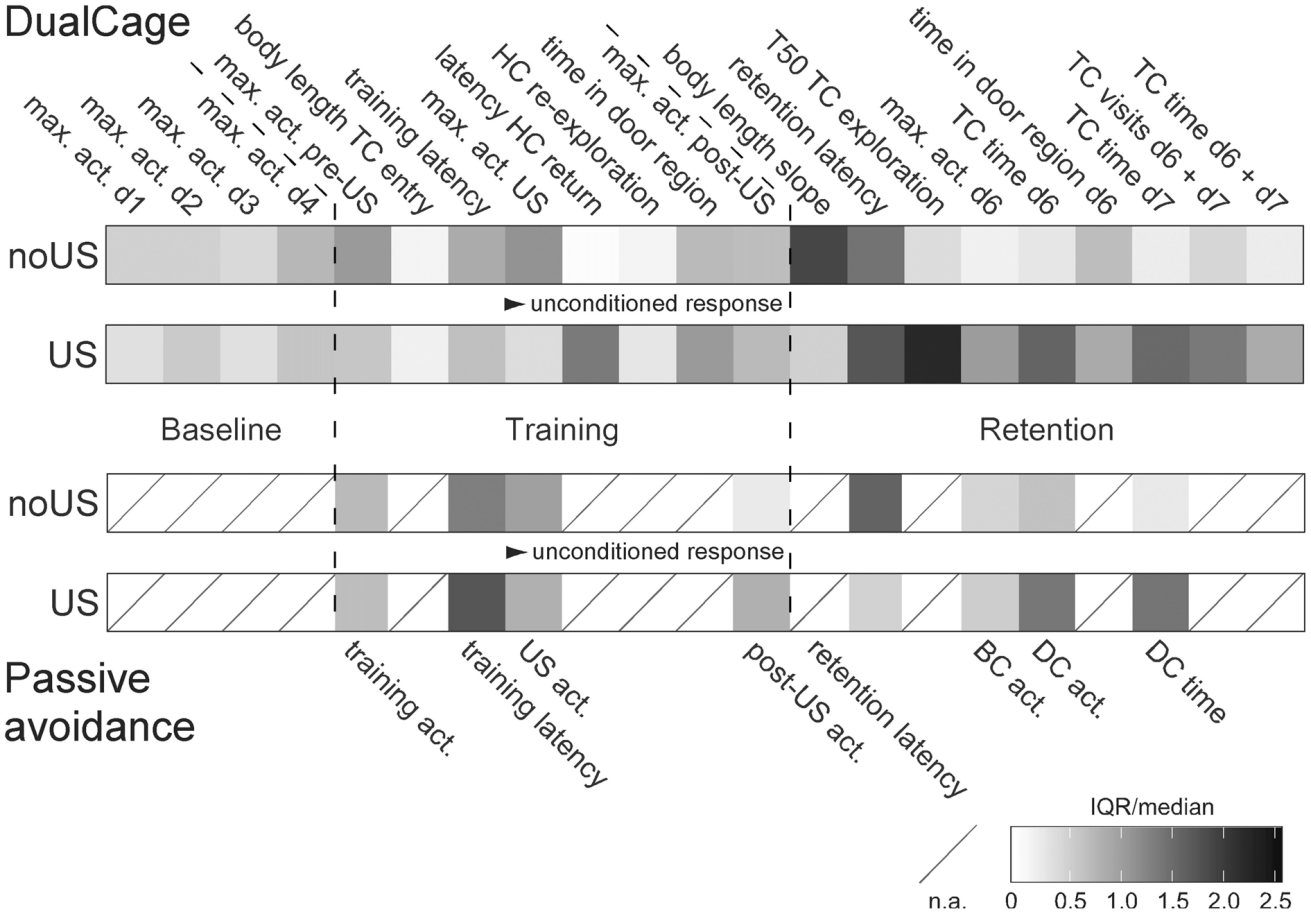
**Increased variation particularly in the retention test measures of shocked C57BL/6J mice in DualCage but not passive avoidance experiments**. Normalized (relative) variation across all DualCage measures (from Figure S5) vs. passive avoidance measures (from Figure S4) in non-shocked (no US) and shocked (US) C57BL/6J mice. Normalized variation was determined by the inter-quartile range (IQR) divided by the median as non-parametric analog of the coefficient of variation. Higher variation occurred in the retention test measures with particularly higher variation in measures from shocked mice in the DC than from shocked mice in passive avoidance. act., activity; BC, bright compartment; d, day; DC, dark compartment; HC, home compartment; max., maximum; TC, test compartment; train., training; US: shock.

In the first 15 min after reopening of the door, non-shocked mice spent considerable time in the TC, while shocked mice generally avoided the TC providing for a significant difference (*U* = 104; *P* < 0.001; Figure 3G). The locomotor activity of shocked mice initially reduced and increased only gradually, while non-shocked mice showed the opposite activity pattern. The statistical differences between the two groups disappeared 2.5 h after the door was reopened (Figure 3H), indicating that the threat of the open door induced a long-lasting suppression of locomotor activity in shocked mice. The time elapsed between opening of the door and the first full entry into the TC (the transfer latency) was short during training and quantified as the time when 50% of the mice entered the TC (*T*_50_ = 64.8 s; Figure 4A). In the retention test, all non-shocked mice entered the TC again with a short delay (*T*_50_ = 21.6 s; Figure 4A). Shocked mice showed substantially increased transfer latencies (*T*_50_ = 1.8 h; Figure 4A) with considerable inter-individual variation. One out of 13 mice did not enter the TC within 48 h. Upon TC entry all non-shocked mice explored the TC completely as indicated by Boolean map analysis (see Figure S1), with low individual variation (Figure 4B) and in a very short time (*T*_50_ ~ 4.4 min; Figure 4F). In contrast, shocked mice showed large inter-individual variation of their progression of TC exploration (Figure 4B). In general, TC time spent in the TC (Figures 4D,G) and entries (data not shown) were largely confined to the dark phase and were significantly correlated (Figure 5). High variation was confined to retention latency and subsequent progression of TC exploration, and was not observed for any other measure in shock-exposed mice (Figure 6).

### Correlation analyses of behavior data in C57BL/6J mice do not predict individual performance differences

Since we observed considerable individual variation among shocked B6J mice, we performed correlation analyses across a number of derived measures to determine whether measures of activity during the first days, and specific training measures, predicted post-shock behavior. For example, no correlation existed between shock activities and transfer latencies in B6J mice (Figure 5). Although many activity measures were significantly correlated across different days (Figure 5), indicating high individual consistency, these measures did not predict whether individual mice would show short or long transfer latencies in the retention tests. Thus, transfer latencies were certainly not merely reflecting general differences in activity. Interestingly, delayed retention latency was significantly correlated with a slower TC compartment exploration during training (Figure 5).

### Irrespective of individual differences, known fear differences remain between substrains

**Figure 7:**
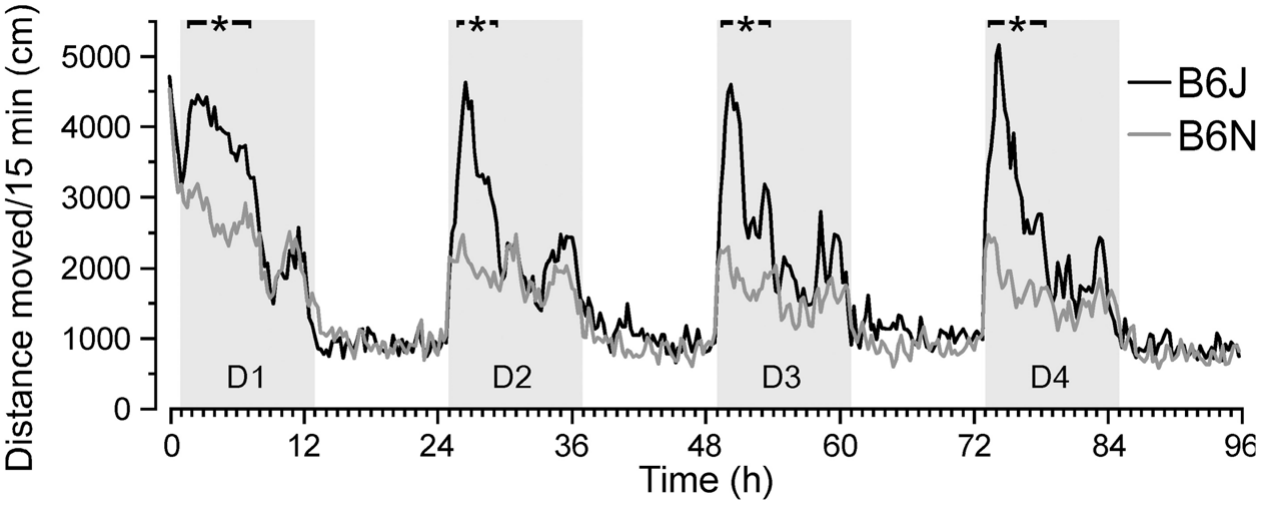
**Substrain-specific differences in circadian activity**. C57BL/6N (B6N) mice showed reduced circadian activity during the initial dark phases of days 1–4 (D1–4) compared to C57BL/6J (B6J) mice. Similar initial activity immediately after placement in the DualCage in the first h of day 1 followed by activity drop of B6N (*n* = 27) but activity increase of B6J mice (*n* = 24). B6N displayed significantly lower activity values [*F*_(1,49)_ ≤ 4.08; ^*^P < 0.05] than B6J mice during the first half of the dark phases on days 1–4 as indicated by black horizontal bars on the top.

To test how individual variation among isogenic mice relates to differences between substrains, we compared the performance of B6J with that of B6N mice. Fear and extinction differences based on freezing have been reported previously between these highly related substrains in classical tests (Stiedl et al., 1999; Siegmund et al., 2005). In both substrains, activity was maximal during the first hour of DualCage exposure (novelty) at the end of the light phase (Figure 7). After the onset of the dark phase, the activity of B6N mice continued to decrease, whereas the activity of B6J mice increased. During all four dark phases of days 1–4 the maximum activity of B6J mice was significantly higher than that of B6N mice. Significant activity differences emerged with a 30-min delay after light offset and lasted for approximately 5 h during the first half of the dark phase (Figure 7).

Despite general differences in locomotor activity, the latencies to enter the TC during training in the naïve state did not differ between B6J (*T*_50_ = 64.8 s) and B6N mice (*T*_50_ = 76.2 s; *P* = 0.328; Figure 4A). Shock-exposure resulted in a similar activity reduction in B6N mice (data not shown) as in B6J mice (Figure 2C). However, shocked B6N mice showed a slower HC re-exploration (Figure S2B) than B6J mice (Figure 2D, Figure S2A) as compared for three specific times (1 h, 6 h, 11 h) after training on day 5 (Figure S2C). There was no difference in the time spent in the door area in the HC on day 5 after training compared to shocked B6J mice (Figure S2D) indicating no avoidance difference before the door was opened. During the 24-h period after training, door approaches in the HC did not differ between shocked B6J and B6N mice but were lower than in non-shocked mice of both substrains with B6N mice showing high variability (Figure S2D).

The latencies to re-enter the TC in the retention test did not differ between non-shocked B6J (*T*_50_ = 21.6 s) and B6N mice (*T*_50_ = 28.8 s; *P* = 0.42; Figure 4A), as expected on the basis of similar training latencies. In contrast, shocked B6N mice showed significantly longer transfer latencies (*T*_50_ = 23.4 h) than B6J mice (*T*_50_ = 1.8 h; *P* = 0.034; Figure 4A). The total time spent in the TC increased similarly in B6J and B6N mice from retention test day 1 to day 2 (Figure S2C) with higher variance in shocked than in non-shocked mice. The increase of explored TC area and TC exploration time served as indices of fear extinction. Non-shocked B6J and B6N mice showed a similarly short halftime (*T*_50_ ~ 4.6 min) to explore the TC after its first entry in the retention test (Figures 4B,C,F). However, in contrast to B6J mice, only 12 out of 15 shocked B6N mice re-explored the TC. These B6N mice showed a delayed TC exploration (Figure 4B) resulting in *T*_50_ ~ 24.8 min (Figure 4F) that was significantly longer than that of non-shocked B6N mice (*P* < 0.0001), but similar to the TC exploration of shocked B6J mice (Figure 4F) with comparable TC time (Figures 4D,E,G) suggesting similar extinction in both substrains.

In conclusion, both B6J and B6N mice exhibit substantial individual differences in their avoidance responses, but this does not preclude the detection of significant fear expression differences, such as increased fear of B6N vs. B6J mice as previously observed.

## Discussion

Here we describe substantial individual differences in avoidance behavior to re-enter the TC during deliberate choice as identified by high-content behavioral monitoring using a new fear learning approach, the DualCage. We show considerable inter-individual variation specifically in long-lasting fear responses based on risk assessment and avoidance. Despite large inter-individual variation, the DualCage discriminated fear responsiveness between two genetically closely related C57BL/6 substrains, similar to classical fear conditioning tests (Radulovic et al., 1998; Stiedl et al., 1999; Siegmund et al., 2005; Bryant et al., 2008).

The DualCage approach combines a number of important advances that in combination are essential for valid high-content behavioral phenotyping. We exploited ethologically valid behavior exclusively motivated by intrinsic novelty-seeking to explore the TC. This intrinsically motivated behavior was unfettered from human intervention and other unspecific stressors. While classical fear learning tests rely on one core measure such as freezing and transfer latency, we used multiple measures to analyze the behavioral performance with a focus on avoidance as reliable and unambiguous emotional measure in the DualCage. To match the test duration with the time scale of the examined physiological processes (Fonio et al., 2012a,b) to determine valid inter-individual variation, the retention test lasted for 48 h instead of 10 min as adhered to in classical tests. We observed substantially extended transfer latencies with a median of ~1.8 h, demonstrating that the standard passive avoidance test duration, with a cut-off time of maximally 10 min (Baarendse et al., 2008), only grasps a small fraction of the dynamic range of the avoidance behavior. Short test durations result in more homogeneous distribution of avoidance performances (Baarendse et al., 2008; Figure 6) probably due to truncated data. The long test duration increased the bandwidth of responses fostering the characterization of individual differences despite their genetic identity, similar experimental history (Caldij et al., 2011) and lack of human interference. This is only possible based on the refined/novel behavior assay development (see also Choi and Kim, 2010), whereas classical tests have a number of shortcomings in measuring and interpreting behavior (Spruijt et al., 2014). Classical tests therefore need complementation by high-content studies as performed here to reduce the interpretational ambiguity.

In many affective disorders, avoidance behavior is a core symptom (American Psychiatric Association, 2013), while freezing is a core measure of emotionality in rodents. Freezing assessment is essential for studies of the fear circuitry (e.g., Maren, 2011) and pharmacological and/or optogenetic modulation of memory processes (e.g., Goshen et al., 2011), but so far has limited symptomatic value in humans as indicated by only a few reports on freezing (e.g., Azevedo et al., 2005; Hagenaars et al., 2014). Avoidance requires higher cognitive functions involving the hippocampus (Ambrogi Lorenzini et al., 1996; Baarendse et al., 2008) and the prefrontal cortex (Barraclough et al., 2004). Inappropriate risk-taking is linked to increased impulsivity and attributed to impaired executive function due to cortical hypofunction (Paulus, 2007). Avoidance is used here as unambiguous analog of the human endophenotype for improved studies of animal models (de Mooij-van Malsen et al., 2009).

Our study indicates the presence of individuality of genetically identical mice (Freund et al., 2013), with the extremes of avoidance and risk assessment behavior, resulting in short or extremely long/absent transfer latencies. This classifies B6J mice as low (bold, pro-active) or high fear (shy, reactive) individuals (Koolhaas et al., 2007), respectively. The absolute variation in transfer latencies and TC exploration of shock-exposed mice exceeded that of any other measure acquired in the DualCage (Figure 6) providing evidence for specifically altered emotional responsiveness. This avoidance variation, serving as index of individuality in coping styles within substrains, clearly suggests epigenetic modulation of fear expression (Wong et al., 2005; Caldij et al., 2011; Zovkic and Sweatt, 2013).

Statistically, the results of this study indeed suggest that the power to detect significant strain- or treatment-dependent differences is reduced due to the observed inter-individual variation, and therefore, larger group sizes are necessary (Button et al., 2013). However, this in turn might also lower the risk of type 1 errors (false positives) potentially reducing the replicability problem (Benjamini et al., 2010; Button et al., 2013).

Individuality in rodents can emerge during development as a consequence of intrauterine position, nutrition and social interaction, imprinting errors, maternal stress and disease, and early postnatal interactions such as handling (Lathe, 2004) and maternal experience (Siegmund et al., 2009). Additionally, random events, such as residual segregation, individual differences in molecular states or changes in the epigenetic state of a genome in general, dynamically interact with each other, so that ontogeny possibly amplifies particular functional consequences in one individual while not in the other. Individual alterations (e.g., in the structure and function of the nervous system) in turn also affect the responsiveness to environmental stimuli (Dias and Ressler, 2014), such as fear responses. Since individual response variation increased here only after aversive experience, this suggests an important role of learning and memory on individuality. Similar to the findings of Siegmund et al. (2009), the prediction of high or low avoidance behavior was not possible on the basis of baseline behavior during habituation in the HC and after contextual fear conditioning (training and consolidation). Such prediction would be extremely valuable to identify individuals at risk for early treatment to counteract the potential etiology of PTSD but may require additional independent measures such as EEG (Machida et al., 2013) and/or heart rate (Stiedl et al., 2009) or cortisol levels as claimed in human studies (Shalev et al., 1998; Yehuda et al., 1998). Isogenic C57BL/6J mice respond to three unconditioned stressors with consistent heart rate response magnitudes further indicating inter-individual fear expression differences on the autonomic level (Liu et al., 2014). Furthermore, we have preliminary evidence for altered avoidance responses depending on rearing/housing conditions (unpublished observation). Thereby, it is possible to bias the avoidance response based on the experiential history (e.g., postnatal stress) of mice to enrich the fraction of PTSD-like responders independent of the genetic background (Molet et al., 2014). However, this is cannot be resolved in this study due to the unknown rearing conditions of mice at the breeder.

Human studies have indicated individual susceptibility differences as important determinant of treatment efficacy in affective disorders (Borsini, 2012). Inter-individual differences in emotional responsiveness serve as translational model for PTSD in humans (Holmes and Singewald, 2013) displaying variation in proneness to stressors (Yehuda and LeDoux, 2007; Daskalakis et al., 2013). Consequently, a full appreciation of epigenetic variation affecting individual responsiveness is imperative for increased validities of animal models on a genetic, molecular, and pharmacological level as ultimate prerequisite for treatment efficacy of personalized medicine.

In contrast to the variation in emotional responsiveness, the activity measures showed low variance within the B6J strain that persisted throughout the retention test. Furthermore, unlike in classical behavior tests (Stiedl et al., 1999), B6N mice covered significantly less distance than B6J mice throughout the first part of the dark phase during habituation (Figure 7), indicating a strong genetic effect (heritability *h*^2^ ~ 0.47 for the 4-h dark phase activity starting 1 h after onset of D2–D4) attributable to few SNP differences between the two substrains that only emerged under these experimental conditions. The low variance of locomotor activity, that even persisted across the retention test, suggests low epigenetic influence on basic behavioral expressions such as locomotion under these experimental conditions.

The variance in avoidance during the retention test observed in B6J mice was also observed in B6N mice with more individuals showing persistent avoidance. However, there was no difference in extinction between B6J and B6N, based on the *T*_50_ measure to explore the TC after its first full entry (Figure 4F). This is inconsistent with fear extinction in classical tests (Stiedl et al., 1999; Siegmund et al., 2005), as replicated by us (Figure S3), and extended to passive avoidance experiments (Figure S4). Fear-conditioned heart rate responses to an auditory cue in B6J and B6N mice resulted in similar extinction rates, when retention tests were performed in the home cage without human intervention (Stiedl et al., 1999). It is plausible that extinction rates are similar between substrains when there is no negative impact of unspecific stressors (handling, novelty) on behavioral performance. Extinction learning might be particularly susceptible to unspecific stressors and generalized fear (Radulovic et al., 1998; Stiedl et al., 1999) resulting in cognitive impairment (Diamond et al., 2007). Our experiments did not provide indications of generalized fear of B6N mice before the TC was accessible during the retention test, whereas these mice exhibited increased fear generalization in classical tests (Radulovic et al., 1998; Stiedl et al., 1999). Thus, generalized fear observed in B6N mice might be a consequence of unspecific stressors such as human handling and novelty in classical fear assays that is expected to affect subsequent emotional and cognitive responses (Diamond et al., 2007). However, we are dealing here with prolonged (2-day) within-session fear extinction, whereas most fear conditioning experiments deal with short (e.g., 3–9 min) within-session extinction combined with across-session extinction (tested once daily for several days). Prolonged fear extinction is more ethologically relevant than short-term across-session extinction as relatively artificial procedure. It remains to be tested whether stronger avoidance responses or signs of generalized fear can be incubated by prolonged periods of consolidation as in fear conditioning (Pamplona et al., 2011) and whether different timing of extinction sessions affects remote avoidance responses as reported in fear conditioning (e.g., Golub et al., 2009) and spontaneous recovery of fear. In general, there is limited information on extinction in operant fear learning tasks (Ögren and Stiedl, 2010) as opposed to classical fear conditioning.

Finally, in some mice the persistent avoidance of the TC for 2 days indicated long-lasting (tonic) rather than transient (phasic) emotional state (Sylvers et al., 2011). While this may be a semantic issue (McNaughton, 2011), conceptually, the open door to the TC serves as specific threat (=fear) rather than being a diffuse threat (=anxiety) as previously defined (Lang et al., 2000; Sylvers et al., 2011). Furthermore, we are convinced that the decision-making process involved in re-entering and subsequent re-exploration of the TC makes this operant conditioning assay, offering animals a choice, distinct from classical fear conditioning. Furthermore, while freezing can be elicited and expressed on a subconscious level (LeDoux, 2014), we here deal with conscious fear and risk assessment based on deliberate choice to enter the TC with the involvement of the prelimbic prefrontal cortex in the expression of conditioned contextual fear (Burgos-Robles et al., 2009; Kim et al., 2013).

In conclusion, we provide evidence for substantial individual differences in fear expression in both isogenic mouse substrains B6J and B6N based on fear responses in the DualCage. Individual avoidance differences emerged only after severe emotional challenge and with long-term persistence indicating its validity as PTSD model (Daskalakis et al., 2013). This study highlights the importance of inter-individual differences on valid and unambiguous interpretations of emotional behavior in genetically identical organisms. High avoidance performances have been implicated in increased susceptibility for anxiety disorders fostering the exploitation of behavioral extremes for disease/disorder modeling. This approach brings a new quality to the study of the phenotype to parallel the study of the genotype (Houle, 2009).

### Conflict of interest statement

The authors declare that the research was conducted in the absence of any commercial or financial relationships that could be construed as a potential conflict of interest.
