# Supplementary material for: Display of individuality in avoidance behavior and risk assessment of inbred mice
Source: Front Behav Neurosci. 2014 Sep 16;8:314. doi: 10.3389/fnbeh.2014.00314 (PMC4165351; doi:10.3389/fnbeh.2014.00314)
Supplement: Supplementary file 2 [file DataSheet1.DOCX]

***Supplementary Material***

**Display of individuality in avoidance behavior and risk assessment of inbred mice**

**Torben Hager^1,2^, René F. Jansen^2,3^, Anton W. Pieneman^2,3^, Suriya N. Manivannan^4^, Ilan Golani^5^, Sophie van der Sluis^2,6^, August B. Smit^3^, Matthijs Verhage^2^, and Oliver Stiedl^2,3,^***

^1^Sylics BV, PO Box 71033, 1008 BA Amsterdam, The Netherlands

^2^Department of Functional Genomics, Center for Neurogenomics and Cognitive Research, Neuroscience Campus Amsterdam, VU University Amsterdam, The Netherlands

^3^Department of Molecular and Cellular Neurobiology, Center for Neurogenomics and Cognitive Research, Neuroscience Campus Amsterdam, VU University Amsterdam, The Netherland

^4^Biobserve GmbH, Siegburger Str. 35, 53757 St. Augustin, Germany

^5^Department of Zoology, Faculty of Life Sciences and Sagol School for Neuroscience, Tel Aviv University, Israel

^6^Department of Clinical Genetics, Neuroscience Campus Amsterdam, VU University Medical Center

***Correspondence:** Oliver Stiedl, Center for Neurogenomics and Cognitive Research, Neuroscience Campus Amsterdam, VU University Amsterdam, De Boelelaan 1085, Room A-062, 1081 HV Amsterdam, The Netherlands

oliver.stiedl@cncr.vu.nl, Tel.: +31 20 5987100, Fax: +31 20 5986968

1. **Supplementary Figures**


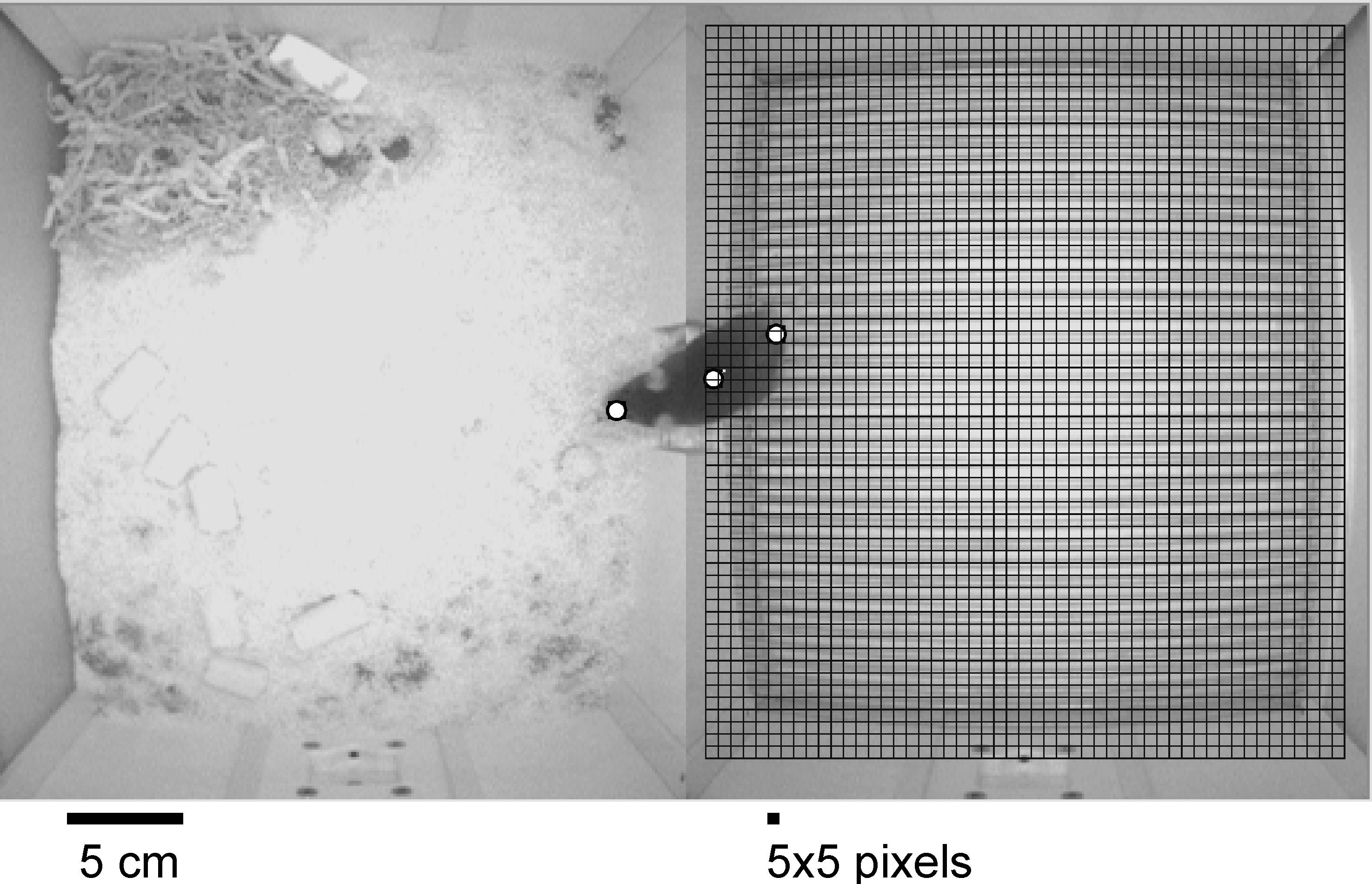


FIGURE S1 | Boolean map of exploration. The matrix of 5 x 5 pixels/zone (exemplified here for the test compartment) was used to quantify the area explored in each compartment (given in % of the total area) based on the nose position of a mouse. Revisiting the same area does not increase the total area. This allows determining the exhaustive exploration of the whole test compartment.


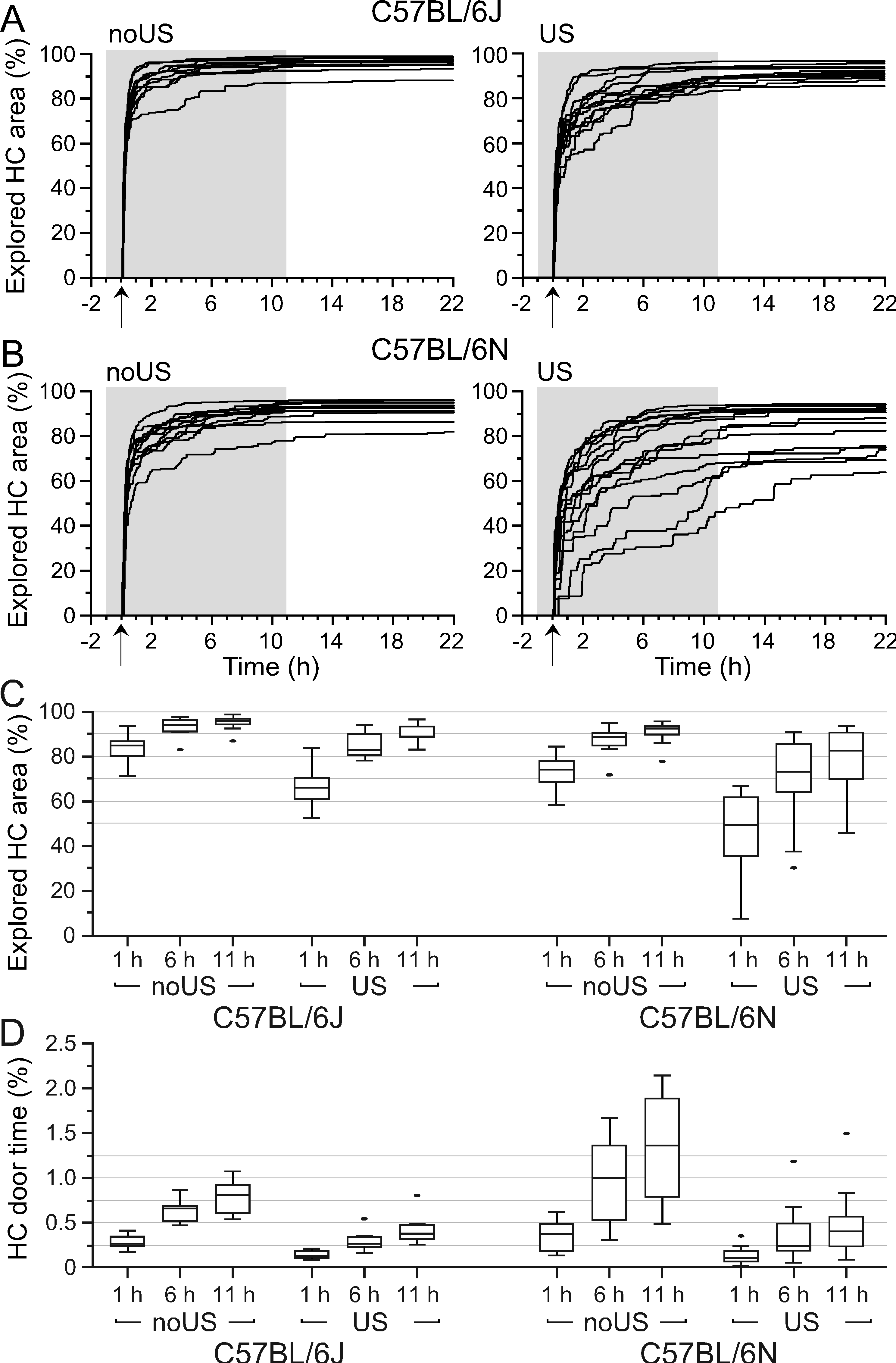


**FIGURE S2 | Home cage re-exploration and door exploration after training in C57BL/6J and C57BL/6N mice.** Upon HC return from the TC after training, there was a slower re-exploration in shocked (US, right panels) than in non-shocked (noUS, left panels) C57BL/6J **(A)** (as shown in Figure 2D**)** and C57BL/6N mice **(B)** as determined by the Boolean map of progressive exploration (Figure S1) with significantly lower maximum explored area. B6N mice did not differ from B6J mice when not exposed to shock (noUS), whereas shocked B6N mice (US) exhibited a profound delay in HC re-exploration compared to B6J mice. **(C)** Box plots show the group comparisons of explored HC area (from A,B) of non-shocked (noUS) and shocked (US) B6J and B6N mice after training at specific times as indicated. **(D)** The cumulative time spent in the door region of the HC after training in the TC show reduced door time in shocked versus non-shocked mice and suggest an increased attractor function in non-shocked C57BL/6N mice.





**FIGURE S3 | Slightly lower fear responses and significantly faster extinction of C57BL/6J versus C57BL/6N mice in context-dependent fear retention tests.** Fear-induced changes were assessed in classical fear conditiong. Mice were subjected to a single shock (closed circles) or no shock (open circles) during training. Fear responses were assessed on the basis of inactivity **(A)**, activity **(B)** and exploration area **(C)** on 4-5 consecutive days (d1-d5) 24 h after training in 180-s test sessions. Delayed extinction was observed in 6N mice. **P*<0.05, ***P*<0.01, ****P*<0.001 between groups. Values indicate mean±SEM, n/group=9-10. The fear conditioning experiments were performed as previously described (Stiedl et al., 1999).


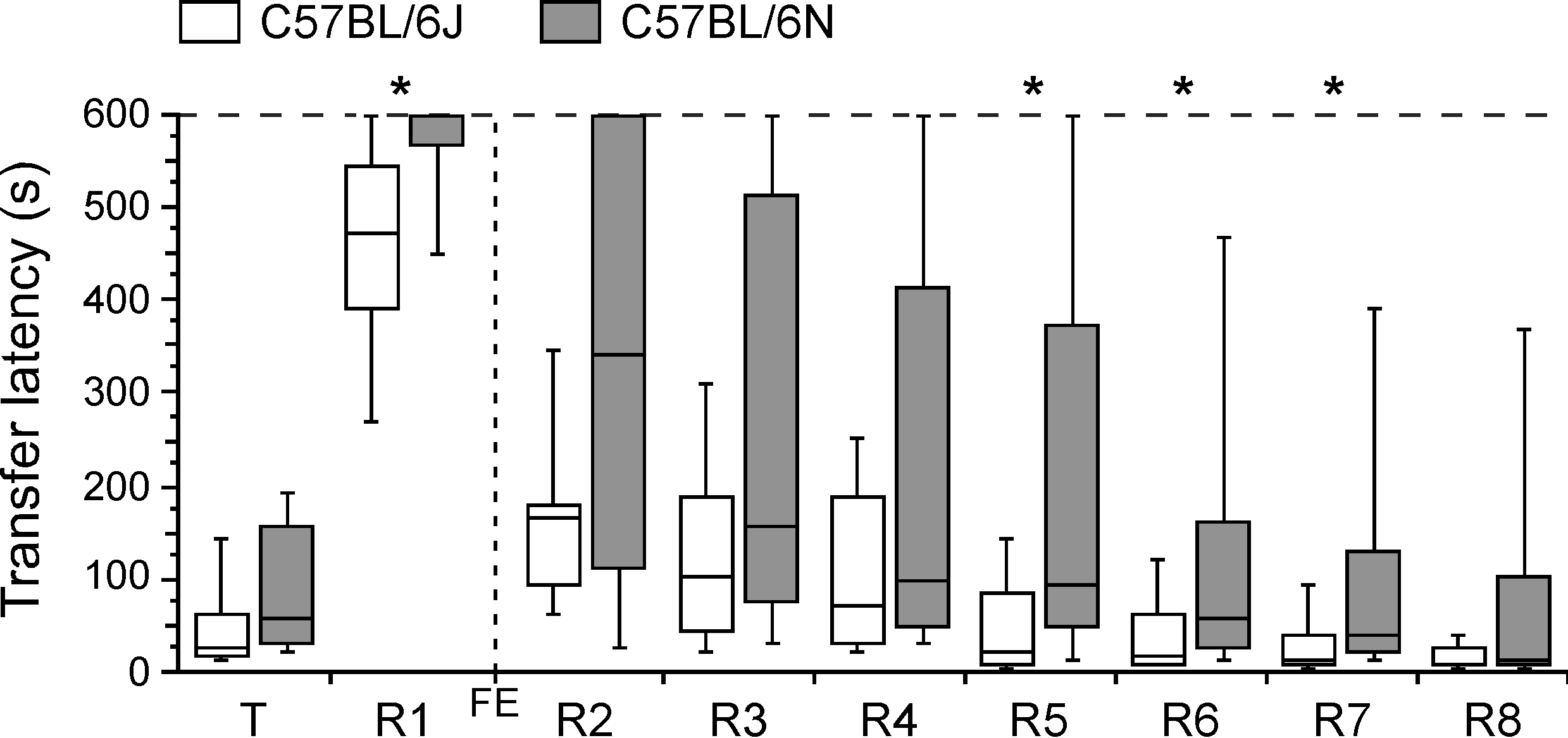


**FIGURE S4 | Lower transfer latencies and faster extinction of C57BL/6J versus C57BL/6N mice in passive avoidance retention tests.** Latencies were assessed in classical passive avoidance experiments with a cut-off time of 600 s. Mice were subjected to a 0.7 mA/2s US (shock) after the first dark compartment (DC) entry during training (T) on day 1. B6N mice showed significantly longer dark compartment transfer latencies than B6J mice during retention test 1 (R1) on day two. On day 3, mice were subjected to a forced exposure (FE) to the dark compartment for a total of 8 min (minus the time spent in the DC during R1). From day 4 on, additional retention tests (R2-R8) were performed at 24-h interval. Mice of both substrains not subjected to US re-entered the DC immediately upon door opening in all retention tests (median transfer latency >25 s). The long upper whiskers of the box plots in B6N mice denote a subpopulation of mice (3 out of 12) that showed substantially delayed DC transfer latencies indicative of delayed fear extinction. Mice were placed in the bright compartment (1000 lx) and the latency to enter the dark compartment (10 lx) was determined. Both compartments were identical in size to that of the DualCage. For detailed passive avoidance information see (Baarendse et al., 2008). n=12/group; **P*<0.05 based on Mann-Whitney U-test.
